# Neuronal Pentraxin 2 Binds PNNs and Enhances PNN Formation

**DOI:** 10.1155/2019/6804575

**Published:** 2019-10-20

**Authors:** Heleen M. Van't Spijker, Dáire Rowlands, Jean Rossier, Barbara Haenzi, James W. Fawcett, Jessica C. F. Kwok

**Affiliations:** ^1^John van Geest Centre for Brain Repair, University of Cambridge, Cambridge, UK; ^2^University of Massachusetts Medical School, Worcester, USA; ^3^Neuroscience Paris Seine, Pierre and Marie Curie University, Paris, France; ^4^Institute of Experimental Medicine CAS, Prague, Czech Republic; ^5^School of Biomedical Sciences, Faculty of Biological Sciences, University of Leeds, Leeds, UK

## Abstract

The perineuronal net (PNN) is a mesh-like proteoglycan structure on the neuronal surface which is involved in regulating plasticity. The PNN regulates plasticity via multiple pathways, one of which is direct regulation of synapses through the control of AMPA receptor mobility. Since neuronal pentraxin 2 (Nptx2) is a known regulator of AMPA receptor mobility and Nptx2 can be removed from the neuronal surface by PNN removal, we investigated whether Nptx2 has a function in the PNN. We found that Nptx2 binds to the glycosaminoglycans hyaluronan and chondroitin sulphate E in the PNN. Furthermore, in primary cortical neuron cultures, the addition of NPTX2 to the culture medium enhances PNN formation during PNN development. These findings suggest Nptx2 as a novel PNN binding protein with a role in the mechanism of PNN formation.

## 1. Introduction

The perineuronal net (PNN) is a mesh-like extracellular matrix (ECM) structure formed on the surface of neurons to regulate plasticity. It appears at the closure of critical periods [1–4]. A critical period is a stage of high plasticity during brain development, when neurons form or prune synapses to consolidate an efficient network [5]. After the closure of these periods the capacity for plasticity is reduced, at which point the PNN is fully formed [3]. The PNN consists of a hyaluronan (HA) backbone [6, 7], to which are bound a variety of chondroitin sulphate proteoglycans (CSPGs), such as aggrecan, versican, brevican, and neurocan [8]. The core proteins of CSPG are decorated with chondroitin sulphate (CS) glycosaminoglycan (GAG) chains [9, 10]. The CSPGs are bound stably to HA by hyaluronan and proteoglycan link proteins (HAPLNs) [8, 11, 12], and the structure is further stabilised by tenascin R binding to the C-termini of the CSPGs [13, 14]. Several enzymes are known to break down the PNN. Both chondroitinase ABC (chABC) [4, 15] and hyaluronidase [16, 17] remove the PNN from the surface of the neurons and induce a renewed capacity for plasticity. For example, when the PNN is removed in the visual cortex, ocular dominance plasticity can be reopened [4].

The PNN regulates plasticity through several pathways [18]. Firstly, the PNN functions as a physical barrier between the neuronal surface and the extracellular space. The PNN buffers ions [19] and protects neurons from oxidative stress [20] and toxic proteins [21]. Secondly, the PNN acts through its binding partners, such as orthodenticle homeobox 2 (Otx2) and semaphorin-3A (Sema3A). Otx2 is known to bind the PNN, after which it is internalized and functions as a transcription factor to regulate the maturation of neurons in the visual cortex [22, 23]. When the PNN is removed, Otx2 is no longer internalized. Sema3A is a chemorepulsive protein [24]. When it is bound to the PNN, it confers an additional inhibition to the PNN and repels approaching axons [24, 25]. Thirdly, the PNN limits lateral diffusion of membrane-bound proteins, such as *α*-amino-3-hydroxy-5-methyl-4-isoxazolepropionic acid (AMPA) receptors [26]. The molecular interactions of the PNN are not yet fully known, and improving the knowledge on PNN regulation by its interacting molecules may allow manipulation of the PNN for plasticity enhancement.

The PNN is attached to only a subset of neurons [7, 27–32]. In the cortex, parvalbumin-positive GABAergic fast-spiking interneurons (PV-INs) are the main group of neurons that possess a PNN [33, 34]. PV-INs receive signals from large groups of neurons [1], and their PNN allows them to regulate plasticity. When CSPGs are digested in the cortex using chABC, electrophysiological measurements showed that inhibitory activity is reduced [35]. The balance between inhibitory and excitatory signals was altered, and the pattern of activity resembled the juvenile state of the cortex [35]. This suggests that PNNs are important for balancing inhibitory and excitatory signals. Thus, manipulation of PNNs might help to restore balance in the brain of patients with plasticity dysfunctions [36], such as epilepsy [37] or schizophrenia [38].

*Neuronal pentraxin 2* (*Nptx2*, also known as *Narp*) is an immediate early gene expressed in the central nervous system [39]. Nptx2 is a calcium-dependent lectin, which accumulates at excitatory synapses where it regulates AMPA receptor mobility [40]. *Nptx2* KO mice are viable and fertile but show a reduction in spontaneous firing rating of PV-INs [41]. Furthermore, in *Nptx2* KO mice, the critical period for ocular dominance plasticity does not close [42]. The role of Nptx2 in critical period closure [42] overlaps with the function of the PNN [1, 4]. Interestingly, it has been shown that treatment with chABC to digest the CSs in the PNN removes Nptx2 from the surface of neuronal dendrites [41]. These findings suggest that Nptx2 may interact with the PNN. Here, we show that Nptx2 binds PNN GAGs and enhances PNN formation. These results suggest that Nptx2 is a potential regulator for PNN formation.

## 2. Materials and Methods

### 2.1. Chemicals

For immunohistochemistry, virus injection, neuronal cultures and Western Blots, the following are used: goat anti-Nptx2 (Santa Cruz, sc-12125), chicken anti-NeuN (Millipore, abn91), rabbit anti-myc-tag (OriGene, TA100029), rabbit anti-parvalbumin (Abcam, ab11427), biotinylated WFA (Sigma-Aldrich, L1516), mouse anti-6X his-tag (Abcam, ab18184), Hoechst (Thermo Fisher, H3570), his-tag NPTX2 protein (R&D Systems, 10889), chABC (Sigma-Aldrich, C3667), test hyase (Sigma-Aldrich, H3506), strep hyase (Sigma-Aldrich, H1136), and lenti-*cmv*-*Nptx2* lentivirus (10^7^ TU/mL, OriGene, MR206833L1V).

### 2.2. Virus Injection

All experiments were conducted in accordance with the United Kingdom Animals (Scientific Procedures) Act (1986). All animals were housed in standard housing conditions in a room with a 12-hour light-dark cycle. The animals were housed in groups of 2-4 animals per cage. The animals were fed *ad libitum* and had unrestricted access to water. To allow for the visualization of Nptx2 in the brain, 1 *μ*L lenti-*cmv*-*Nptx2-myc* lentivirus (CMV promoter, DDK-myc tag, 10^7^ TU/mL) (OriGene) was injected in the right somatosensory cortex (AP: -1, ML: 1.5, DV: -1) of five 3-month-old female Listar hooded rats. Rats were first anaesthetised with isoflurane after which the virus injection was performed at a rate of 500 nL/2 minutes. After 18 days, the rats were sacrificed and perfused.

### 2.3. Immunohistochemistry

Rats were perfused with 4% paraformaldehyde (PFA), tissues dissected and postfixed at 4°C for 24 hours in 4% PFA. The brain tissue was then cryoprotected in a 30% sucrose (Thermo Fisher) in PBS. The tissue was then sectioned on a cryostat (Leica CM 3050S) into free-floating sections at 20 *μ*m thickness, which were preserved in PBS with 0.1% *w*/*v* sodium azide at 4°C. To stain the tissue slices, they were first washed 3 times in PBS and then put in blocking buffer containing 3% horse serum, 0.1% bovine serum albumin (BSA), and 1% Tween-20 in 1X PBS. Subsequently, incubation of primary antibodies took place for 16 hours at 4°C. Primary antibodies are as follows: 1 : 100 rabbit anti-myc-tag (OriGene), 1 : 200 anti-NeuN (Millipore), and 1 : 250 WFA (PNN marker, Sigma-Aldrich). After another three washes with PBS-T, incubation of appropriate secondary antibody at a concentration of 1 : 500 mixed with 1 : 10000 Hoechst (Thermo Fisher) in 1X PBS took place for one hour at RT. After three washes with 1X Tris-sodium chloride buffer (TNS), the slices were mounted on slides (twin frost glass slides, Thermo Scientific). To preserve the signal, FluorSave Reagent (Calbiochem) was applied to all slices and left to dry for 24 hours.

### 2.4. Neuronal Dissections and Cultures

Embryos from a Sprague Dawley rat were collected at embryonic day 18. Their heads were harvested and preserved in Hanks' balanced salt solution (HBSS). Cortices were dissected and treated with papain to allow for the separation of neurons. DNAse (Sigma-Aldrich) was applied to remove free DNA, after which neurons were washed with HBSS and filtered (Thermo Fisher, 40 *μ*m cell strainer). The neurons were plated at 120,000 per imaging dish (Greiner Bio-One, 8.7 cm^2^ surface), which had been coated with poly-D-lysine (Sigma-Aldrich). The neurons were maintained in culture by replacing half of the medium (consisting of 100 *μ*L 200 mM L-glutamine (Gibco), 800 *μ*L GS21 (AMS Biotechnology Limited, Abingdon, UK) in 40 mL neuralQ medium (Life Technologies)) every week. To investigate the effect of NPTX2 on the PNN, the medium was enriched with 40 ng/mL NPTX2 (R&D Systems, human *NPTX2* expressed and purified) from 14 days *in vitro* (DIV) onwards. To investigate the effect of PNN removal in the presence of exogenous NPTX2 on the neuronal surface, neurons were treated with chABC (Sigma-Aldrich, 0.2 U/mL final concentration) in 100 mM ammonium acetate at pH 8.0 or Streptomyces hyaluronidase (Sigma-Aldrich, 60 U/mL in 0.002 M final concentration) in sodium acetate at pH 6.0, 1 : 10 diluted in medium. Half of the neuronal medium was replaced with the enzyme mixture, and the cells were placed back in the incubator at 37°C to incubate the enzymes for 30 minutes. Neurons were fixed with 4% PFA for 15 minutes to allow for staining. The neurons were stained with biotinylated WFA (Sigma-Aldrich) 1 : 250, mouse anti-his-tag antibody (Abcam) 1 : 100, rabbit anti-parvalbumin antibody (Abcam) 1 : 250, and Hoechst (Thermo Fisher) 1 : 10000. The imaging was performed on a confocal microscope (Leica DM 600B) at 20x.

### 2.5. Automated Analysis of PNNs with R

Images were captured on a confocal microscope (Leica). The settings used were as follows: 1024 × 1024 format, xyz acquisition, speed at 400 Hz, no pinhole, and no bidirectional imaging. Four lasers were used: 405 nm, 43% open, and 1004 gain for Hoechst staining; 488 nm, 45% open, and 593 gain for WFA staining; 532 nm, 46% open, and 1020 gain for Nptx2-myc tag staining; 635 nm, 42% open, and 946 gain for NeuN staining. Individual PNNs (*n* ≈ 20) were automatically extracted from microscope images (×40, taken on a single confocal plane) using a novel methodology developed in R. In this method, images were uploaded in R in .tiff format. The script, based on Rowlands et al., identifies the PNN circular shapes in the WFA channel, after which it switches to the Hoechst channel to control whether the identified circle was around a cell [43]. For individual PNNs, the area in which the PNN is WFA positive is selected. Subsequently, the mean intensity for the WFA staining in the selected area is measured. The script compares this intensity with the intensity in the comparison channel, in this case the channel for the myc-tag, in the same region. The intensity in both channels was compared to investigate correlation between intensity in the WFA and the myc-tag channel.

### 2.6. Sequential Buffer Treatment to Isolate PNN Molecules

Homogenized 3-month-old rat brains were treated as described in [3]. In short, after homogenization with a Potter Elvehjem homogenizer to preserve cellular integrity, brains were treated with sequential buffers to dissolve molecules from the ECM (buffer 1 : 50 mM Tris buffer saline with 2 mM ethylenediaminetetraacetic acid at pH 7.0) and the cytoplasm and membrane (buffer 2 : 0.5% Triton X-100 in buffer 1), molecules contained by ionic interactions (buffer 3 : 1 M NaCl in buffer 2), and PNN molecules (6 M urea in buffer 2), sequentially. The sequential buffer treatment ensures that the ECM molecules, cytoplasm and membrane molecules, and the molecules contained with ionic interactions are removed before purification of PNN molecules takes place. The samples were treated to preserve proteins with protease inhibitors (Roche) or GAGs with pronase (Roche) followed by TCA and diethyl ether purification.

### 2.7. Western Blot

The protein quantities in the protein samples were determined with a bicinchoninic acid kit (Thermo Fisher), and equal protein quantity of each sample was run on an 4-12% SDS-PAGE gel (Invitrogen) at 120 V and transferred to a PVDF membrane (Invitrogen). 400 ng of NPTX2 protein (R&D Systems) was run as a positive control. After transfer, the blots were probed overnight at 4°C with 1 : 200 goat anti-NPTX2 antibody (Santa Cruz).

### 2.8. ELISA

After purification of the samples with the sequential buffer treatment, the GAG concentration in each sample was quantified with cetylpyridinium chloride (CPC) turbidimetry [44]. Pure samples of individual GAGs were purchased from Seikagaku. Each GAG was biotinylated to a final concentration of 0.02 mg/mL GAG. The amount of biotin was quantified for each sample to ensure equal biotinylation. Nptx2 protein was incubated on plates with high binding capacity (Santa Cruz) for 16 hours, after which the GAGs were incubated. Streptavidin alkaline phosphatase was allowed to bind to the biotin groups on the GAGs. The quantity of bound biotin was determined with para-nitrophenyl phosphate, catalyzed by the phosphatase to produce phenolate of which the wavelength was measured at 405 nm.

### 2.9. GENSAT and Allen Brain Atlas

The Gene Expression Nervous System Atlas (GENSAT) was used to explore the expression pattern of Nptx2. Bacterial Artificial Chromosome engineering was applied to express an enhanced green fluorescent protein (EGFP) gene upstream of the Nptx2 gene. Images were taken from the database available at http://www.gensat.org/GeneProgressTracker.jsp?gensatGeneID=2078. The Allen Brain Atlas was used to explore *in situ* hybridization images of Nptx2 on male mice at p56 (Nptx2-RP_051214_01_H10–coronal, © 2010 Allen Institute for Brain Science, Allen Human Brain Atlas, available from http://human.brain-map.org.).

### 2.10. scRNA-Seq in Visual Cortex

Violin representation of scRNA-Seq in 14236 single cells from the visual sensory primary cerebral cortex in mice 56 days old. Data are from the Allen Institute (file reference VISp_14236_20180912). On the top 13 subclasses are 4 GABAergic (Lamp5, Vip, Sst, and Pvalb), 8 glutamatergic (layer 2/3 IT IntraTelencephalic excitatory neurons, layer 4 excitatory neurons, layer 5 IT, layer 6 IT, layer 5 PT Pyramidal Tract excitatory neurons, NP Near Projecting excitatory neurons, layer 6 CT corticothalamic excitatory neurons, layer 6b excitatory neurons), and 1 astrocyte in various colors according to the code color of Tasic et al. [45]. On vertical left genes detected; Gad2 marker for interneurons; Slc17a7 marker for excitatory neurons; Gaj1 marker for astrocytes; Lamp (lysosomal-associated membrane protein family, member 5), Vip (vasoactive intestinal polypeptide), Sst (Somatostatin), Pvalb (Parvalbumin) four markers delineating four subclasses of interneurons: Sema3a, Sema3e, Nptx1, and Nptx2. Median values are black dots, and values within rows are normalized between 0 and the maximum expression value for each gene (right edge of each row) and displayed on a log10 scale.

### 2.11. Statistics

The analysis of the intensity of the PNN staining in the brain was performed in R with a previously published methodology [43]. The correlation between the intensity of the WFA channel and the myc-tag channel was tested with Spearman's correlation test. The intensity of both channels was measured as a mean intensity for the PNN identified in the WFA channel (*n* ≈ 20, on 5 animals in total). The analysis of the PNN staining on cultures was performed by measuring the PNN surface area and intensity in ImageJ, after which the Nptx2-treated neurons were compared with the untreated neurons in Origin Pro (*N* = 20). The ELISA results were analysed in Origin Pro with a one-way ANOVA (*N* = 3, each ELISA repeated three times).

## 3. Results

### 3.1. Localization of Nptx2 to PNNs

Previous literature suggests that Nptx2 may mediate its action through interaction with PNNs [41]. However, currently in the literature, there is no direct evidence demonstrating an interaction between Nptx2 and PNNs. To investigate this potential link between them, we set out to localize Nptx2 in the adult rat brain. However, none of the commercially available antibodies gave consistent results that could clearly be differentiated from background. In order to examine the subcellular localization of Nptx2 in the brain and to circumvent the problems encountered with the antibodies, we used lentiviral injections to overexpress Nptx2 protein tagged with c-myc in the cortex of adult rats.

**Figure 1:**
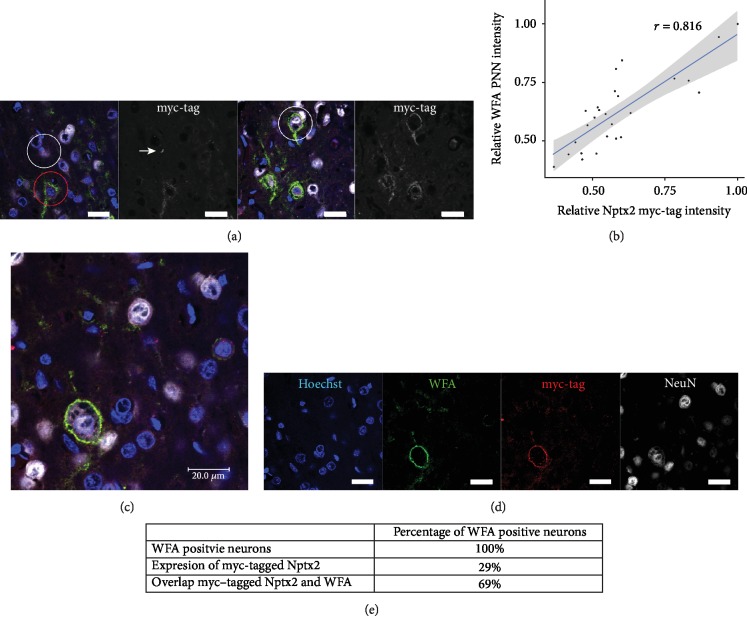
Colocalization of myc-tagged Nptx2 and PNNs. 3-month-old rats were injected with lenti-cmv-Nptx2 into the somatosensory cortex to express myc-tagged Nptx2. Perfused tissue was cut in 20 *μ*m sections and stained with 1 : 250 WFA to identify PNNs, 1 : 100 myc-tag antibody to identify the myc-tagged Nptx2 protein expressed by the lenti-cmv-Nptx2, and 1 : 200 NeuN antibody to identify neurons. (a) Representative images of the somatosensory cortex stained for WFA (green), myc-tag (red), and Hoechst (blue). In the left image, the white circle shows a neuron with intracellular myc-tagged Nptx2, but not on the cell membrane. The red circle shows a neuron with myc-tagged Nptx2 intracellularly and overlapping with the PNN. The white arrow points at the dotty myc-tag staining which is suggestive of vesicle staining. In the right image, the white circle shows a neuron which contains myc-tagged Nptx2 colocalizing with the PNN but shows no signs of intracellular myc-tagged Nptx2. (b) A correlation graph for the intensity of the myc-tag channel and the WFA channel is presented. A correlation analysis of WFA staining intensity and myc staining intensity revealed a correlation of 0.816 which indicates a high level of intensity correlation of the two channels in the PNN area. (c, d) The WFA staining for PNNs (green) is overlapping with the myc-tagged Nptx2 staining (red). (e) An overview of the number of neurons which are expressing the myc-tagged Nptx2 and which are positive for myc-tagged Nptx2 staining on their PNN. Intracellular myc-tag signal-positive neurons and PNN myc-tag signal-positive neurons were calculated. All images were taken in the somatosensory cortex (scale bar = 20 *μ*m).

Rats were injected with the lenti-*cmv*-*Nptx2-myc* at 3 months of age, and their brains were stained 18 days post injection to locate the expressed myc-tagged Nptx2 by staining with an anti-myc antibody. We injected the lenti-*cmv*-*Nptx2-myc* into the somatosensory cortex since there is extensive PNN literature in this region [32, 46]. There were two main patterns of myc-tagged Nptx2 staining. The pattern of the anti-myc staining varied across neurons: some neurons were positive for myc-tag colocalization with WFA in the typical appearance of PNNs and also contained intracellular dotty vesicular-shaped staining, while other neurons had myc staining on their PNNs but no signs of intracellular staining (Figure 1(a)). It is possible that the dotty vesicle staining was caused by endosomal processes as well as vesicles. There were also many cells which had the intracellular vesicular-shaped pattern but showed no Nptx2 staining in the PNN (Figure 1(a) white circle on left image). Of the PNN-positive neurons (determined by WFA), 66% were positive for myc staining on their PNN, and 29% showed the intracellular vesicle-shaped myc staining pattern (Figures 1(d) and 1(e)). Within cells, there was a particulate staining with a vesicular appearance (Figure 1(a), white arrow, and Figure 1(c)). Around cells, the myc staining surrounded cells and proximal dendrites (Figure 1(c)). This staining colocalized with the PNN marker WFA and was never found in neurons without WFA staining. The colocalization between the extracellular myc-tag staining and the WFA staining was quantified with an in-house PNN analysis program written in R [43]. A correlation coefficient of 0.816 was found, which indicates that the myc-tagged Nptx2 indeed locates to the PNN on neurons (Figure 1(b)). These data show that myc-tagged Nptx2 partially associates with PNNs and that the Nptx2 diffuses from or gets secreted by Nptx2-producing cells to PNN-bearing cells where it binds or is sequestrated.

**Figure 2:**
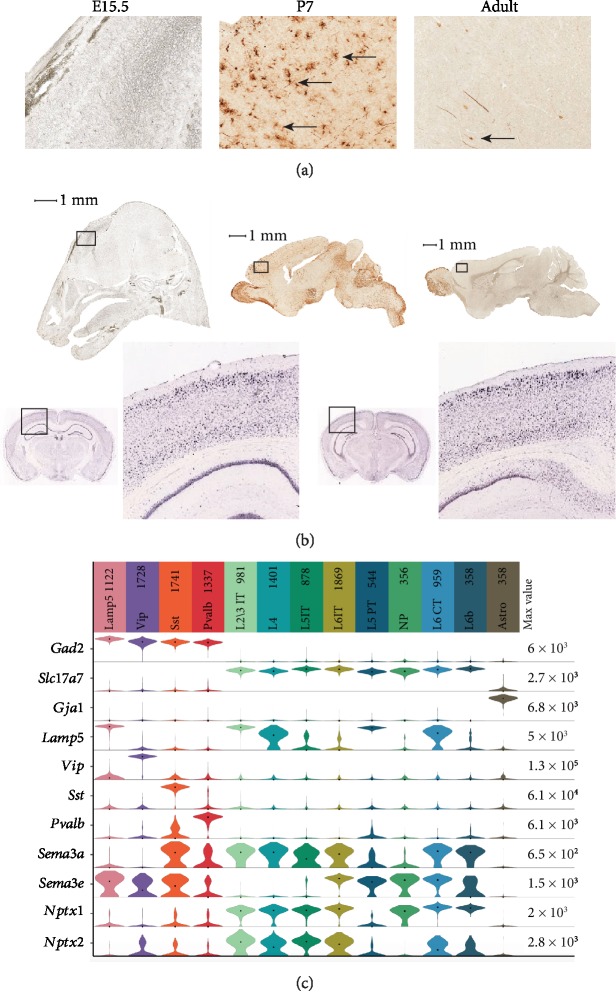
Nptx2 is expressed in the cerebral cortex. (a) These images were taken from the Gene Expression Nervous System Atlas. Bacterial Artificial Chromosome engineering was applied to express an EGFP gene upstream of the *Nptx2* gene. High expression of *Nptx2* was found in the cerebral cortex of mice at P7. Low expression was found in the cerebral cortex of adult mice, at P42. Low to no expression was found in the cerebral cortex of mice at E15.5. These findings indicate that expression of *Nptx2* is high during perineuronal net development, after which it is reduced. The black arrows indicate neurons which express *Nptx2* (scale bar = 1 mm). (b) These images were taken from the Allen Brain Atlas. The images were produced with in situ hybridization for *Nptx2* on male mice at p56. They show that expression of *Nptx2* is present in adult mice in the cortex: Nptx2-RP_051214_01_H10–coronal, © 2010 Allen Institute for Brain Science, Allen Human Brain Atlas, available from http://human.brain-map.org. (c) Violin representation of scRNA-Seq in 14236 single cells from the visual sensory primary cerebral cortex in mice 56 days old. Data are from the Allen Institute (file reference VISp_14236_20180912). On the top 13 subclasses are 4 GABAergic (Lamp5, Vip, Sst, and Pvalb), 8 glutamatergic (layer 2/3 IT IntraTelencephalic excitatory neurons, layer 4 excitatory neurons, layer 5 IT, layer 6 IT, layer 5 PT Pyramidal Tract excitatory neurons, NP Near Projecting excitatory neurons, layer 6 CT corticothalamic excitatory neurons, layer 6b excitatory neurons), and 1 astrocyte in various colors according to the code color of Tasic et al. [45]. On vertical left genes detected; Gad2 marker for interneurons; Slc17a7 marker for excitatory neurons; Gaj1 marker for astrocytes; Lamp (lysosomal-associated membrane protein family, member 5), Vip (vasoactive intestinal polypeptide), Sst (Somatostatin), Pvalb (Parvalbumin), four markers delineating four subclasses of interneurons: Sema3a, Sema3e, Nptx1, and Nptx2. Median values are black dots, and values within rows are normalized between 0 and the maximum expression value for each gene (right edge of each row) and displayed on a log10 scale.

To investigate the regional expression of Nptx2, it was necessary to visualize *Nptx2* expression in the brain. Due to the lack of a good Nptx2 antibody for immunohistochemistry, we sought to use the online Gene Expression Nervous System Atlas (GENSAT) [47] to investigate *Nptx2* expression. GENSAT is an NIH-funded, publicly available gene expression atlas of the mouse central nervous system. The protocol applied to provide the images for the atlas is a Bacterial Artificial Chromosome (BAC) engineering system. An EGFP gene is inserted upstream of the ATG start codon of the gene of interest. This method allows for the visualization of cells which express the gene of interest. We investigated the expression of *Nptx2* in the cortex with the GENSAT database. *Nptx2* is expressed by neurons in the cortex of the mouse at P7 (Figure 2(a)). Expression of *Nptx2* is still present in the cortex at adult age in reduced quantities. In the Allen Brain Atlas [48], *in situ* hybridization images of adult male mice are available (Figure 2(b)) for visualizing RNA expression directly. In the Allen Brain Atlas images, *Nptx2* is expressed in the adult mouse cortex, with a high level in layer 2 (Figure 2(b)). An RNA sequencing experiment measured levels of *Nptx2* in the cortex. It was found that *Nptx2* is mostly expressed by intratelencephalic neurons in the cortex as well as corticothalamic neurons, vasoactive intestinal peptide (VIP), and somatostatin- (SST-) expressing neurons (Figure 2(c)). Interestingly, to this date, these neurons are not known to form PNNs. However, since Nptx2 is secreted by neurons, it would be possible for Nptx2 to diffuse through the ECM to bind to PNNs on neighbouring neurons. Here, the role of PNNs could be acting as a local “chelator,” preventing Nptx2 from further dispersion through the cortex. Interestingly, the idea that a PNN-binding molecule can be produced in one place then diffuse to another place in order to bind to PNNs has been previously described for the transcription factor Otx2. Beurdeley and colleagues showed that Otx2 is produced and secreted from the choroid plexus, travels to the visual cortex, binds to the disulphated CS-E sugars in CS-GAGs, and affects PNN maturation locally [23, 49]. Nptx2 might also diffuse from one neuron to another, over shorter distances. The combined results from Figures 1 and 2 suggest that *Nptx2* is expressed (Figure 2) and secreted in the cortex by a variety of excitatory neurons and interneurons, which allows Nptx2 to diffuse and reach PNN-positive neurons (Figure 1).

### 3.2. Binding of Nptx2 to PNN Components

**Figure 3:**
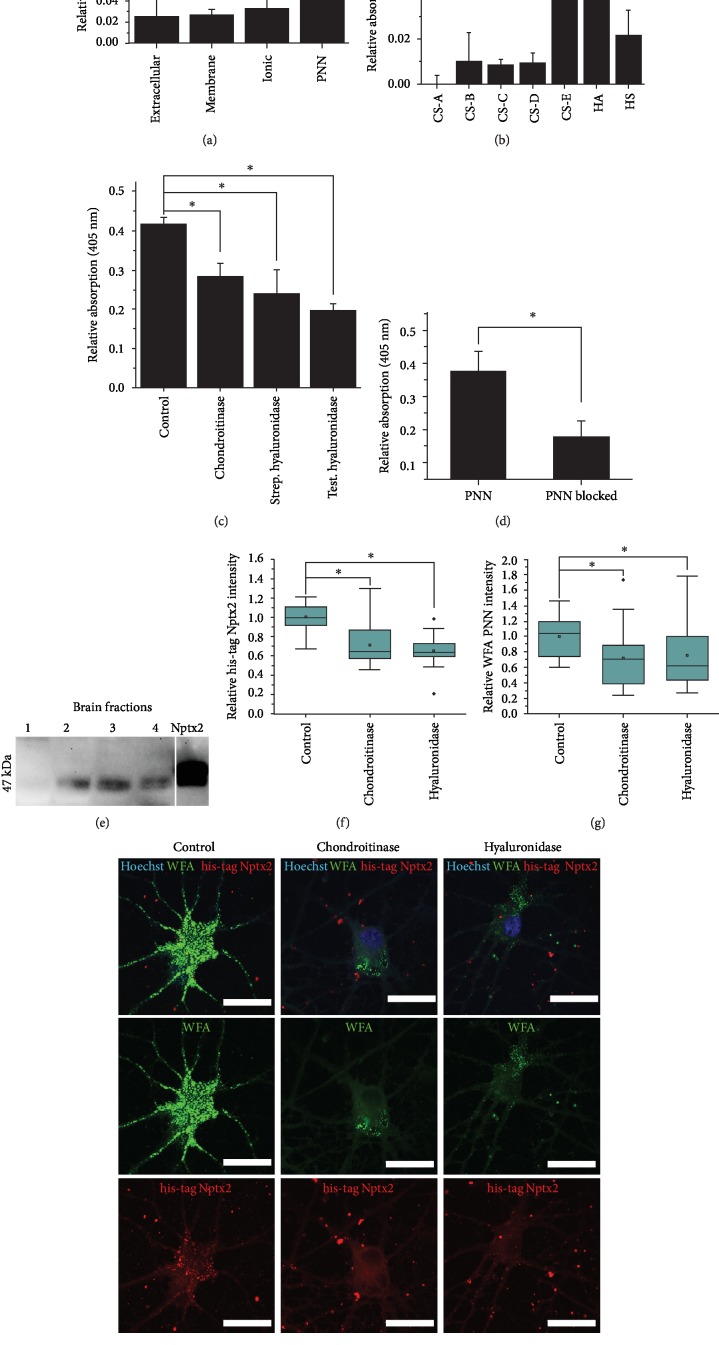
Nptx2 binds to the PNN. (a) Sequential buffer treatment of homogenized brain tissue was applied to purify the GAGs in the four separate samples. The GAGs were randomly biotinylated to allow for detection with streptavidin alkaline phosphatase on a custom-made NPTX2 ELISA plate. NPTX2 binds preferentially to PNN GAGs (*p* = 2.38*E* − 4). (b) To investigate which PNN GAGs can bind NPTX2, an ELISA was performed with biotinylated samples of purified GAGs. The applied GAGs were chondroitin sulphates (CS) A, B, C, D, and E, hyaluronan (HA), and heparan sulphate (HS). NPTX2 binds significantly to HA and CS-E. ELISA results were analysed with ANOVA (*p* = 2.01*E* − 4). (c) To investigate which of the PNN GAGs are responsible for the binding of NPTX2 to PNN GAGs, an NPTX2 ELISA bound by the PNN GAG fraction was treated with the enzymes chondroitinase ABC, Streptomyces hyaluronidase, and testicular hyaluronidase for one hour. All these enzymes were able to significantly reduce the signal of the bound PNN GAGs, which indicates that all enzymes break GAG bonds responsible for the binding of PNN GAGs to NPTX2. This result indicates that both, CS-E and HA in the PNN GAG sample, bind to NPTX2 on the plate (*p* = 2.21*E* − 4). (d) To confirm whether PNN glycan binding to NPTX2 could be blocked in a competition ELISA, one set of wells was treated with unbiotinylated HA before exposure to biotinylated PNN glycans. The hyaluronan indeed blocked the PNN glycans from binding (*p* = 0.008). (e) Sequential buffer treatment of homogenized rat brain tissue was applied to purify extracellular molecules (1), membrane molecules (2), molecules contained by ionic interactions (3), and PNN molecules (4). The samples were run on a Western blot, alongside a commercial NPTX2 control sample, at 120 V on a 4-12% gel. Nptx2 was found in samples 2, 3, and 4. This result suggests that Nptx2 is located on the membrane, contained by ionic interactions and in the PNN. (f–h) Cortical neurons from E18 rat brains were cultured to reach 35 DIV. Neurons were treated with chondroitinase ABC or Streptomyces hyaluronidase for 30 minutes. Neurons were fixed with 4% PFA for 20 minutes. After blocking for 1 hour, neurons were incubated with WFA and his-tag antibody to stain for the his-tagged NPTX2. Appropriate secondary antibodies were applied, and three wash steps were performed after each step. Representative images of neurons are shown (h). The relative Nptx2 his-tag intensity (f) and WFA PNN intensity (g) were significantly reduced after treatment with chondroitinase ABC or Streptomyces hyaluronidase (*n* = 20, ANOVA, *p* = 5.24*E* − 8*p* = 0.039, resp.). These findings indicate that both enzymes successfully removed the PNN from the neuronal surface and this removal of the PNN resulted in a removal of the exogenous Nptx2 protein from the neuronal surface (scale bar = 20 *μ*m).

In order to bind to PNNs after its secretion from distant cells, Nptx2 must bear a specific binding affinity for certain PNN component(s). We next sought to investigate the structures in PNNs which enable the binding of Nptx2. Most of the known PNN binding proteins have been shown to bind to the CS-GAG chains in the PNN [23, 24]. We therefore first investigated whether Nptx2 binds to the various forms of GAGs present in PNNs. PNN molecules were purified from rat brain homogenates using a sequential buffer purification method [3, 9]. This method allows for the separation of molecules from the different compartments of the ECM (1) buffer soluble, (2) detergent soluble, (3) charge-bound, and finally (4) stable urea-soluble fraction that contains mostly PNN molecules (fractions 1-4). These fractions were investigated by Western blots probed with anti-Nptx2 antibody. Bands corresponding to Nptx2 (47 kDa) were observed in fractions 2, 3, and 4, suggesting the presence of Nptx2 on neuronal membranes and in the PNN fraction (Figure 3(e)). This finding implies that Nptx2 is produced in the adult CNS and that at least some of it is tightly bound to PNNs.

In order to confirm the binding of Nptx2 to PNN components, we developed a glycan-ELISA using purified NPTX2 (expressed from the human *NPTX2* gene). GAGs were isolated from different brain fractions and conjugated with biotin. Human NPTX2 was immobilised at the bottom of the ELISA plates; biotinylated GAG chains isolated from different brain fractions were allowed to bind. The results showed that NPTX2 preferentially binds the fraction 4 GAGs which come predominantly from PNNs, but much weaker to GAGs isolated from other fractions (Figure 3(a)). To confirm whether this result was due to binding of NPTX2, unbiotinylated HA was incubated on immobilised NPTX2 before incubation of biotinylated PNN glycans. This competition ELISA showed binding of PNN glycans was significantly decreased after HA incubation (Figure 3(d)). These results suggest that NPTX2 binds the PNN by interacting with PNN GAGs.

Our previous work showed that semaphorin-3A, another PNN binding molecule, binds to PNN via CS-E GAG chains [24]. To investigate which GAG chains are responsible for the binding of NPTX2, the NPTX2 ELISA was repeated with pure GAG samples from commercial sources, with predominant sulphation patterns, including CS-A to CS-E, HA, and heparan sulphates (HS). The results showed that NPTX2 binds with high affinity to CS-E and HA (Figure 3(b)) and with a weaker affinity to HS than to CS-B, CS-C, and CS-D. There is no binding to CS-A. These findings indicate that NPTX2 binds the PNN preferentially through CS-E and HA.

Next, we further characterised which GAG chains bind to NPTX2, by digesting the bound PNN GAGs with different GAG-digesting enzymes, including a CS-digesting enzyme, chondroitinase ABC (chABC), a HA-digesting enzyme, Streptomyces hyaluronidase (strep hyase), and a CS/HA-digesting enzyme, testicular hyaluronidase (test hyase). While chABC and strep hyase provide specific digestions of CS or HA, respectively, test hyase digests both HA and CS groups and could provide insights if both GAGs are involved. We first bound NPTX2 to the plates, then allowed the PNN GAGs to bind to the NPTX2, after which the GAG-digesting enzymes were applied. The results showed that an enzymatic cleavage by chABC, test hyase, and strep hyase resulted in a significant reduction of signal of the bound PNN GAGs (Figure 3(c)). This suggests that the interaction between NPTX2 and PNN GAGs is mediated by both CSs and HA.

The binding of NPTX2 to the PNN was further investigated *in vitro* using E18 cortical neuronal cultures. Cultures were matured until 35 DIV. NPTX2 with a his-tag was added to the medium to allow for visualization of potential binding of NPTX2 in the culture. NPTX2 was observed on the surface on neurons, forming a typical punctate PNN pattern *in vitro* (Figure 3(h), control). To confirm the involvement of HA and CS in the binding, the GAGs were then cleaved enzymatically on live cultures with chABC or strep hyase. Treatment with chABC and strep hyase reduced PNN staining on the neuronal surface (Figures 3(g) and 3(h)). At the same time, the staining for the his-tag Nptx2 on the neuronal surfaces was also significantly reduced (Figures 3(f) and 3(h)). These results indicate that exogenous NPTX2 binds to PNNs and can be removed from the neuronal surface by enzymatic digestion of CS and HA. Digesting either the CS chains and the HA chains is sufficient to significantly reduce his-tag NPTX2 staining from the PNN, which indicates that these GAG chains are responsible for the binding of NPTX2 to the neuronal surface.

### 3.3. Nptx2 and PNN Formation

**Figure 4:**
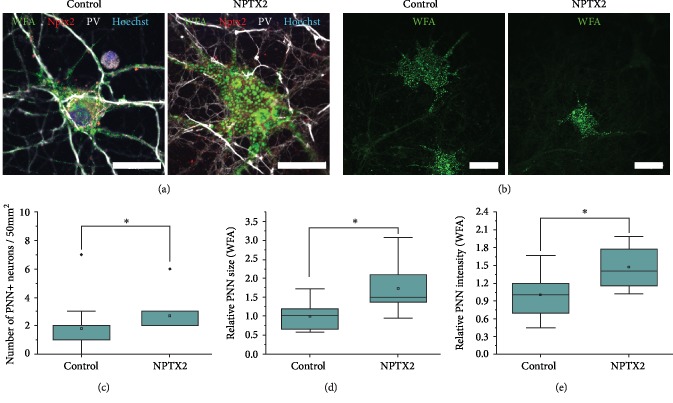
NPTX2 enhances PNN formation. (a) Cortical neurons dissected from E18 rat brains were treated with NPTX2 in their standard neuronal medium from 14 DIV until 28 DIV. Neurons were fixed with 4% PFA and stained with WFA combined with parvalbumin (pv) antibody and his-tag antibody. (a) Representative images of PNN-positive neurons are shown (63x, scale bar = 20 *μ*m). (b) Lower magnification images (20x, scale bar = 20 *μ*m). (c) The amount of PNN-positive neurons/50 mm^2^ was quantified and compared between cultures treated with NPTX2 or not. The number of PNN-positive neurons was significantly higher in cultures treated with NPTX2 (*p* = 0.019). This result indicates that at 28 DIV NPTX2 treatment has increased the number of PNN-positive neurons. For each PNN-positive neuron, the relative size (d) and intensity (e) of their PNN were measured by WFA PNN staining and analysed in ImageJ. It showed significant increase in PNN size in NPTX2-treated cultures (*p* = 4.72*E* − 7) and significant increase in WFA intensity in NPTX2-treated cultures (*p* = 3.93*E* − 7). These results indicate that NPTX2 treatment caused neurons at 28 DIV to be further along in the development of a fully grown PNN (Student's *t*-test).

Nptx2 has previously been shown to regulate AMPA receptor mobility [40], and *Nptx2* KO mice demonstrate persistent plasticity in the visual cortex [42]. In order to investigate if Nptx2 binding to nascent PNNs is related to PNN formation and maturation, we cultured E18 cortical neurons in the presence of NPTX2 (14-28 DIV) until their maturation, a stage when PNNs start to enwrap the surface of neurons and can be visualized by WFA staining. We and others have previously mapped out the developmental profile of PNNs in cortical neurons in culture [50]. PNNs began to show a punctate pattern between 14 and 21 DIV and continued to mature >45 DIV. We thus started the NPTX2 treatment at 14 DIV, a stage prior to PNN development. The neurons were imaged at 28 DIV because PNN formation has not yet been completed at that time in control neurons [50]. Treatment of neurons with 40 ng/mL NPTX2 significantly enhanced the number of PNNs on neurons in the culture at 28 DIV (Figures 4(a)–4(c)). The relative PNN size, measured by area, was also significantly increased when stained with WFA (Figures 4(a), 4(b), 4(d), and 4(e)). The amount of PNN-positive PV-INs was quantified to investigate the maturation level of the PV-INs. In control cultures, 25-35% of PV-INs had started to form a PNN at 28 DIV, while in NPTX2-treated cultures 35-45% of PV-INs had started to form a PNN, which suggests PV-INs were more mature after NPTX2 treatment. These results indicate that NPTX2 enhances PNN formation in cultured neurons.

## 4. Conclusion

The aim of this study was to examine the association of Nptx2 with PNNs; we identified the binding partners of Nptx2 in PNNs and examined the effect of Nptx2 on PNN formation. To study the possible association of Nptx2 with PNNs *in vivo*, we injected a lentivirus expressing myc-tagged Nptx2 into the brain of 3-month-old rats and visualized its location. This method was chosen since it was not possible to stain for endogenous Nptx2. Transfection efficiency was high, and Nptx2 appeared to be localized intracellularly. Nptx2 was also observed to colocalize with WFA-positive PNNs. Some neurons which showed colocalization of Nptx2 with PNNs also contained intracellular Nptx2; however, there were also neurons showing colocalization of Nptx2 with PNNs without intracellular expression of Nptx2. This suggests that Nptx2 can diffuse through the ECM from cells that produce it and then bind to PNNs. The colocalization was quantified with a new automated R script demonstrating a very close anatomical association between Nptx2 and PNNs [43]. Furthermore, the correlation between the WFA staining and the staining for the myc-tag is suggestive of a quantitative correlation between the PNN and Nptx2: the denser the PNN the more Nptx2 can bind. These results suggest that Nptx2 spreads through the cortex after it is secreted by neurons and binds to PNNs.

Next, we analysed the binding mechanism of Nptx2 to PNNs. We fractionated brain homogenate into four compartments by the application of the sequential buffer treatment purification method [3, 9]. This method separates buffer-soluble, membrane-associated, charge-bound, and PNN matrix molecules. Nptx2 was found in the membrane-associated fraction, the charge-bound fraction, and the PNN fraction.

To identify to which PNN molecules Nptx2 can bind, an ELISA was performed to measure binding to the GAGs from the four ECM compartments. The GAGs were investigated since other PNN binding proteins were found to bind to the GAG chains in the PNN [23, 24] and Nptx2 can be removed from the neuronal surface with chABC [41]. NPTX2 bound preferentially to PNN GAGs, with much lower binding to the GAGs from the other fractions. To investigate the type of GAG bound by NPTX2, we performed enzymatic cleavage on PNN fraction glycans with chABC, strep hyase, or test hyase. These enzymes reduced NPTX2 binding, suggesting that the molecule binds to both CS and HA. These results show that NPTX2 binds to the PNN and the binding depends on the PNN GAGs. The same ELISA was then used to measure NPTX2 binding to purified glycans, demonstrating strong binding to both CS-E and HA.

In order to investigate NPTX2 binding to PNNs, we added tagged NPTX2 to mature cortical neuronal cultures (which produce PNNs). The NPTX2 associated with WFA-positive PNNs in culture. Enzymatic treatment with chABC and strep hyase showed that both enzymes remove WFA staining for PNNs and also his-tag NPTX2 staining from the neuronal surface. These findings indicate that the tested enzymes successfully reduce the HA and CS chains in the PNN, and this reduction leads to the removal of NPTX2 from the neuronal surface.

A possible effect of NPTX2 on the formation of the PNNs was investigated in cortical neuronal cultures. When the medium was enriched with NPTX2, more neurons formed PNNs by 28 DIV and the PNN on these neurons was larger than on control neurons. These findings indicate that NPTX2 enhances PNN formation. This is not in accordance with the findings presented by [51] that PNNs are unaltered in *Nptx2* KO mice. Pelkey et al. showed that there were no alterations in WFA staining in the adult hippocampus of *Nptx2* KO mice. However, since this experiment was performed in adult mice only, it is possible that an altered development of PNNs in the *Nptx2* KO animals was missed in this study. It would be interesting to investigate whether *Nptx2* KO mice have altered PNN development at P7, when the PNN is starting to be formed and *Nptx2* expression peaks.

From the current findings, it cannot be concluded through which molecular pathway NPTX2 enhances PNN formation. Interestingly, another PNN binding protein, Otx2, also enhances PNN formation [23]. Otx2 binds to the PNN, and after it enters the neuron, it enhances PNN formation [23]. Otx2, similar to Nptx2, is not synthesized by the PNN-bearing neurons but is synthesized instead in the choroid plexus [23]. Nptx2 is also not synthesized by PNN-bearing PV-INs: Nptx2 is expressed in the cortex by a variety of interneurons and excitatory neurons. Since Nptx2 expression is dependent on neuronal activity [39], it is possible that the activity of excitatory neurons leads to Nptx2 expression which in turn leads to PNN maturation on the nearby interneurons. Synapses are regulated by the PNN, and both the PNN and Nptx2 are known to be able to regulate AMPA receptor mobility [26, 40, 41]. The interaction of Nptx2 with PNNs may provide a control mechanism for the regulation of cortical activity by PV-INs. Further investigation is needed to test this hypothesis.

We identified in this study Nptx2 as a PNN binding partner which enhances PNN formation. The binding of Nptx2 and the PNN provides a new insight into the regulation of PV-INs in the cortex by Nptx2. Nptx2 is known to have a role in PV-IN maturation and critical period regulation [41, 42, 51]. Here, we presented findings indicating a role for Nptx2 in the maturation of PV-INs and PNN condensation on the neurons.

## Data Availability

The data that support the findings of this study are available from the corresponding author, Heleen van ‘t Spijker, upon reasonable request.

## Abbreviations

PNN: Perineuronal net
Nptx2: Neuronal pentraxin 2
chABC: Chondroitinase ABC
Strep hyase: Streptomyces hyaluronidase
Test hyase: Testicular hyaluronidase
AMPA: *α*-Amino-3-hydroxy-5-methyl-4-isoxazolepropionic acid
KO: Knockout
PV-IN: Parvalbumin interneuron
CS: Chondroitin sulphate
HA: Hyaluronan
Otx2: Orthodenticle homeobox 2
Sema3A: Semaphorin-3A
DIV: Days in vitro
GAG: Glycosaminoglycan.
